# Transcriptome-wide organization of subcellular microenvironments revealed by ATLAS-Seq

**DOI:** 10.1093/nar/gkaa334

**Published:** 2020-05-18

**Authors:** Danielle A Adekunle, Eric T Wang

**Affiliations:** 1 Department of Molecular Genetics & Microbiology, UF Genetics Institute, Center for NeuroGenetics, University of Florida, USA; 2 Department of Biology, Massachusetts Institute of Technology, USA

## Abstract

Subcellular organization of RNAs and proteins is critical for cell function, but we still lack global maps and conceptual frameworks for how these molecules are localized in cells and tissues. Here, we introduce ATLAS-Seq, which generates transcriptomes and proteomes from detergent-free tissue lysates fractionated across a sucrose gradient. Proteomic analysis of fractions confirmed separation of subcellular compartments. Unexpectedly, RNAs tended to co-sediment with other RNAs in similar protein complexes, cellular compartments, or with similar biological functions. With the exception of those encoding secreted proteins, most RNAs sedimented differently than their encoded protein counterparts. To identify RNA binding proteins potentially driving these patterns, we correlated their sedimentation profiles to all RNAs, confirming known interactions and predicting new associations. Hundreds of alternative RNA isoforms exhibited distinct sedimentation patterns across the gradient, despite sharing most of their coding sequence. These observations suggest that transcriptomes can be organized into networks of co-segregating mRNAs encoding functionally related proteins and provide insights into the establishment and maintenance of subcellular organization.

## INTRODUCTION

Subcellular organization is critical for compartmentalization of intracellular processes and spatiotemporal control of RNA metabolism and protein translation. RNAs distribute to distinct microenvironments such as the ER (1,2), the leading edge of the cell (3), axons (4), and dendrites (5).These patterns facilitate cellular functions (6), including cell fate determination (7), directed movement (8), embryonic patterning (9), and synaptic plasticity (10). RNAs can be localized by RNA binding proteins (RBPs), via formation of ribonucleoprotein (RNP) particles or RNA transport granules that may travel on cytoskeleton (11). For example, zipcode-binding protein localizes β-actin mRNA to the leading edge of fibroblasts (12), and the She2/She3/Myo4 complex localizes Ash1 mRNA to budding yeast tips (13). *Cis*-elements unique to each mRNA, even at the isoform level, control the repertoire of RNA binding proteins (RBPs) that they recruit. For example, constitutive or alternative 3′ UTRs of mRNAs can recruit specific RBPs that influence both RNA and protein fate (14–16). Indeed, different RNPs can influence formation of granules or compartments with differing physical properties (17–19) and these properties could play a role in dictating their final destinations. One potential reason to co-distribute RNAs is to facilitate efficient co-translation or co-assembly of the proteins they encode. While extensive efforts have been focused on mapping interactions between *cis*-elements and *trans*-factors (20), a major challenge remains to characterize how RNAs are distributed across different types of RNPs and whether they may be localized to distinct subcellular microenvironments.

Many techniques have been developed to study subcellular localization of RNA. *In situ* hybridization (21) offers high accuracy and resolution, especially with single molecule approaches, but is generally low throughput. To address this limitation, techniques such as MERFISH (22) and FISSEQ (23) have been developed to simultaneously visualize thousands of RNAs. In spite of these advances, *in situ* approaches do not easily reveal physical or biochemical properties of the subcellular compartments to which these RNAs localize. Without super-resolution or expansion microscopy, it can be challenging to determine whether RNAs are associated with structures such as membranes, vesicles, or the cytoskeleton. Proximity labeling techniques using BirA or APEX (24), coupled to deep sequencing, have provided alternative routes towards identifying these associations. However, it is challenging to apply these techniques to tissues *in vivo*, and they require exogenous introduction of fusion proteins to biotinylate specific organelles. Traditional biochemical fractionation is therefore an attractive alternative to separate RNPs with distinct biophysical properties (25). Sedimentation across density gradients have been used to stratify protein complexes across cellular compartments (26) and analyses of sedimentation profiles reveal differences that are typically hidden from both image-based and enrichment-based methods. Fractionation combined with sequencing has been used to analyze the transcriptome of specific cellular compartments that are purifiable (27,28), but this approach has not been used to analyze transcriptomes of many cellular compartments simultaneously with high resolution.

Here, we describe ‘Assigning Transcript Locations Across Sucrose-Sequencing’ (ATLAS-Seq), a detergent-free method that fractionates tissue homogenate across a continuous sucrose gradient by density ultracentrifugation, followed by RNA sequencing and mass spectrometry. We have used this approach to develop a map of the subcellular organization of the transcriptome in mouse liver and find that transcripts encoding proteins involved in similar biological processes display similar sedimentation profiles. These profiles reflect a wide array of cellular compartments and correlate with RBP sedimentation patterns, making predictions about regulatory associations. Global characterization of these profiles is a first step towards the elucidation of how RNA–protein interactions generate and maintain these subcellular compartments.

## MATERIALS AND METHODS

### Subcellular fractionation

Wild-type FVB female mouse livers were dissected and washed in ice cold PBS. Tissue was placed in a tube containing 0.25 M buffered sucrose solution, 20mM Tris, water supplemented with protease inhibitor cocktail and 10 mM ribonucleoside-vanadyl complex (VRC) as a ribonuclease inhibitor) with 2.8 mm ceramic beads and placed in a bead homogenizer to homogenize tissue. Homogenized tissue was centrifuged at 5000 × g for 10 min to remove nuclei. A Biocomp Gradient Master™ was using to generate an 11 ml 10–50% sucrose gradient (with 10 mM VRC). Homogenate was layered onto the gradient, and components were resolved by ultracentrifugation in an SW41 rotor for 3 h at 30 000 rpm (4°C). Twenty-four 0.5 ml fractions were collected from the gradient using the BioComp Piston Gradient Fractionator™. Fractions were split for RNA and protein extraction. RNA was extracted from each fraction by Direct-zol RNA miniprep kit. Ten equivalents of EDTA (relative to the VRC concentration) were added to each sample in Trizol-reagent before ethanol was added to remove the ribonucleoside-vanadyl complex. Protein concentrations were measured by the Pierce BCA protein assay kit.

### RNA-Seq

The Kapa stranded RNA-Seq with RiboErase kit was used for prepare libraries according to manufacturer's instructions. An equal mass (500 ng) of RNA was used as input to each individual library. Libraries quality was assessed using a BioAnalyzer (Agilent, Santa Clara, CA, USA) and quantified using a Qubit (Life Technologies) prior to pooling for sequencing. Pooled libraries were 75-bp paired-end sequenced on an Illumina Next-Seq 550 v2.

### Mass spectrometry

Proteins were reduced with 10 mM dithiothreitol for 1 h at 56^o^ C and then alkylated with 55 mM iodoacetamide for 1 h at 25^o^C in the dark. Proteins were digested with modified trypsin at an enzyme/substrate ratio of 1:50 in 100 mM ammonium bicarbonate, pH 8.9 at 25^o^C overnight. Trypsin activity was halted by addition of acetic acid (99.9%) to a final concentration of 5%. Peptides were desalted using C18 SpinTips (Protea, Morgantown, WV, USA) and then vacuum centrifuged. Peptide labeling with TMT 10-plex was performed per manufacturer's instructions. Lyophilized samples were dissolved in 70 μl ethanol and 30 μl of 500 mM triethylammonium bicarbonate, pH 8.5, and the TMT reagent was dissolved in 30 μl of anhydrous acetonitrile. The solution containing peptides and TMT reagent was vortexed and incubated at room temperature for 1 h. Samples labeled with the ten different isotopic TMT reagents were combined and concentrated to completion in a vacuum centrifuge.

Peptides were separated by reverse phase HPLC (Thermo Easy nLC1000) using a precolumn (made in house, 6 cm of 10 μm C18) and a self-packed 5 μm tip analytical column (12 cm of 5 μm C18, New Objective) over a 140-min gradient before nanoelectrospray using a QExactive mass spectrometer (Thermo). Solvent A was 0.1% formic acid and solvent B was 80% MeCN/0.1% formic acid. The gradient conditions were 0–10% B (0–5 min), 10–30% B (5–105 min), 30–40% B (105–119 min), 40–60% B (119–124 min), 60–100% B (124–126 min), 100% B (126–136 min), 100–0% B (136–138 min), 0% B (138–140 min), and the mass spectrometer was operated in a data-dependent mode. The parameters for the full scan MS were: resolution of 70 000 across 350–2000 *m/z*, AGC 3e^6^ and maximum IT 50 ms. The full MS scan was followed by MS/MS for the top 10 precursor ions in each cycle with an NCE of 32 and dynamic exclusion of 30 s. Raw mass spectral data files (.raw) were searched using Proteome Discoverer (Thermo) and Mascot version 2.4.1 (Matrix Science). Mascot search parameters were: 15 ppm mass tolerance for precursor ions; 15 mmu for fragment ion mass tolerance; two missed cleavages of trypsin; fixed modifications were carbamidomethylation of cysteine and TMT 10-plex modification of lysines and peptide N-termini; variable modifications were methionine oxidation.

### Read mapping, expression analysis, and isoform quantitation

Reads were aligned using Spliced Transcripts Alignment to a Reference (STAR) algorithm (29). RNA-Seq reads were quantified, pseudo-aligned to an mm10 Refseq index, and counted as transcripts per million (TPMs) using the Kallisto quantification program (30). For mitochondrial RNAs reads were pseudo-aligned to an Ensembl mm10 index and the TPM counts for annotated mitochondrial-encoded RNAs from the resulting Kallisto tpm table was used to plot the distribution of mitochondrial-encoded RNAs across the ATLAS-Seq gradient (Figure 3B). RefSeq and Ensembl TPM tables can be found in Supplementary Table S2. The Mixture of Isoforms (MISO) (31) program was used to quantitate alternative isoforms. Only isoforms with <0.2 confidence interval across all fractions were analyzed.

### GO analysis

Data release from AmiGO 2 version: 2.5.12 was used to determine GO enrichments (32). Panther GO enrichment analysis (33) was used to determine GO enrichments for all analyses in the paper with one exception. *P*-values were determined by Fisher's exact test with Bonferroni correction for multiple testing. In Figure 4C, GOrilla (34) was used to determine cellular component GO enrichment categories for single lists. The lists for Figure 4C were ranked from highest to lowest Pearson correlation for positive association and from lowest to highest for negative correlation. GOrilla computed an uncorrected *P*-value according to the HG model and the FDR *q*-value was corrected using the Benjamini-Hochberg method.

### Comparing ATLAS-Seq to ribosome profiling

Ribosome profiling was performed in mouse liver cells (35). Fastq files for ribosome profiling and RNA-Seq in mouse liver were downloaded from NCBI (GEO Accession GSE67305) and processed by Kallisto (30). Fastq files for ribosome profiling performed in HEK293T cells were downloaded from NCBI (GEO Accession GSE65778) (36). Fastq files for TRIP-Seq polysome profiling performed in HEK293T cells were downloaded from NCBI (GEO Accession GSE69352). For Figure 2, weighted counts from polysome sequencing or ATLAS-Seq were calculated similarly to Floor *et al.* (37):

ATLAS-Seq or polysome sequencing weighted counts = $\mathop \sum \limits_{i\ {\rm{ = \ }}x}^y i{n_{\rm{i}}}$

For ATLAS-Seq, *x* = 3 and *y* = 24. For polysome sequencing, *x* = 1 and *y* = 7. Essentially, TPMs were weighted by the fraction number, e.g. ${n_{\rm{i}}}$ is the TPM count in the $i$th fraction, where $i$is the fraction number.

### smiFISH and probes

smiFISH was performed according to (38). 3D Z-stacks were captured by epifluorescence using a Zeiss LSM880 using a 63× 1.4 NA objective and an Axiocam MRm camera. Cy3- or Cy5-conjugated Y flaps were used as secondary probe detectors for all primary probes. All probes and flaps produced and purchased from Integrated DNA Technologies (IDT) following protocols as listed in (38). All primary probe sequences are provided in Supplementary Table S4. NIH 3T3 cells were grown on chamber slides (Lab-Tek) in 10% FBS DMEM media. For smiFISH in liver, wild-type FVB mouse livers were cryosectioned into 7 uM sections and then subjected to the smiFISH protocol. DAPI staining was used to identify nuclei and all coverslips were mounted with Vectashield.

### RBP analysis

RBPs were defined using publicly available datasets of previously characterized RBPS (39,40). Overlap between these datasets and our list of peptides obtained from our mass spectrometry identified 148 RBPs in our mass spectrometry dataset. The peptide profile of each RBP was correlated with the mean profile of RNAs in each ATLAS-Seq RNA cluster.

### Quantification and statistical analysis

Graphs were generated using Matplotlib version 2.2.2. Statistical Analyses were performed using Python, SciPy 1.1.0 and NumPy 1.14.3 libraries. Statistical parameters, statistical tests, and statistical significance (*P* value) are reported in the figures and their legends. Two independent, biological replicate gradients were generated from mouse liver. Each replicate was analyzed independently, with ‘gradient 2’ being the replicate used for all main figures. For hierarchical clustering analysis, the SciPy.cluster.hierarchy library was used. All Correlations were calculated using NumPy corrcoef function which returns a Pearson correlation coefficient for variables. Wilcoxon rank-sum tests were used to compute statistical significance.

## RESULTS

### Detergent-free sucrose fractionation of liver lysate separates RNA signatures by their cellular microenvironments

**Figure 1.**
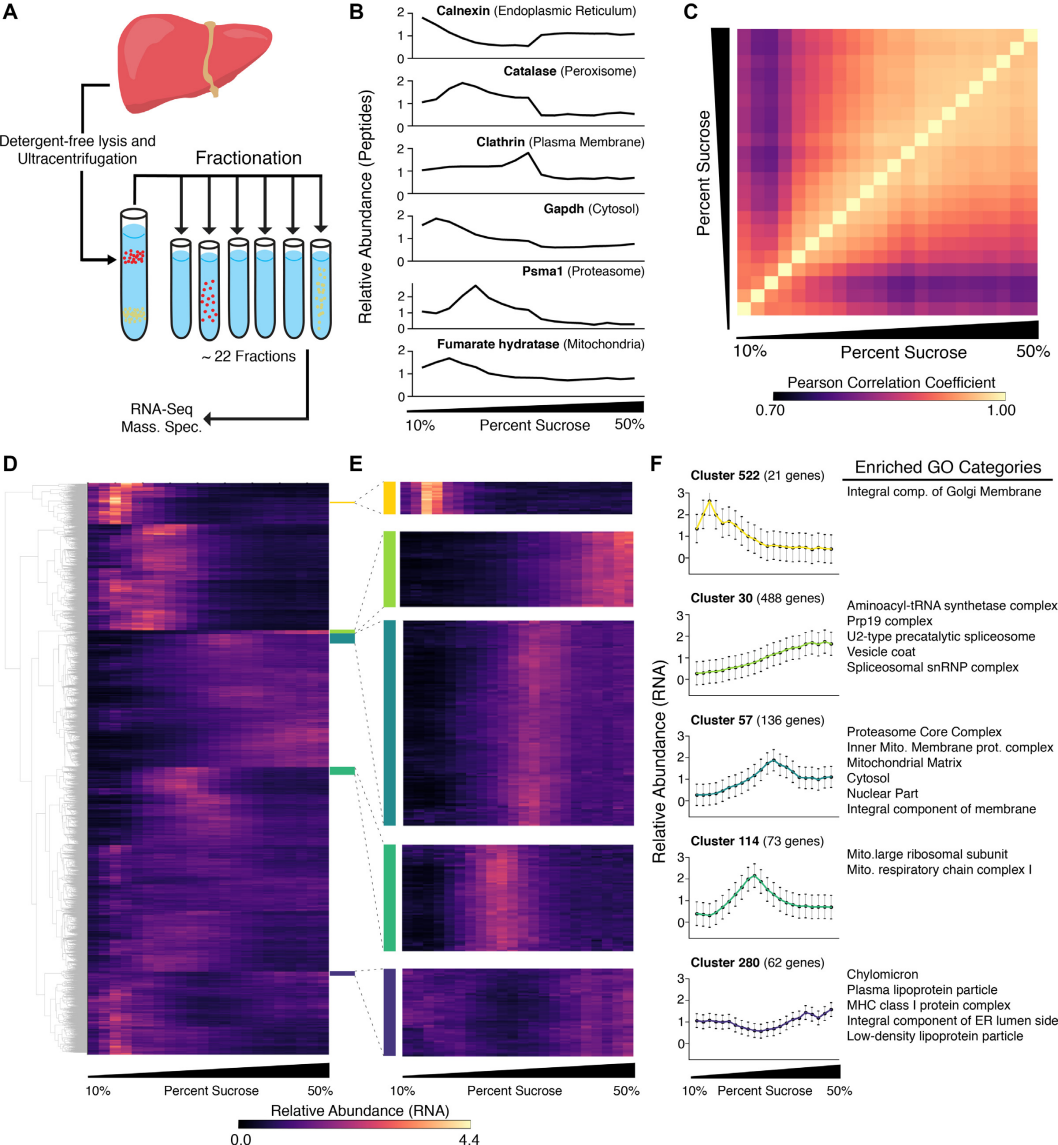
ATLAS-Seq generates transcriptome- and proteome-wide profiles across a density centrifugation gradient. (**A**) Schematic of ATLAS-Seq procedure from mouse liver homogenate depleted of nuclei. (**B**) Relative protein abundance across a single ATLAS-Seq gradient for specific protein organelle markers as assessed by mass spectrometry. (**C**) Heatmap showing Pearson correlation coefficients of gene expression between all pairs of sucrose fractions from a single ATLAS-Seq gradient. (**D**) Heatmap of relative gene expression across a single ATLAS-Seq gradient, organized by hierarchical clusters, where rows are genes and columns are successively denser sucrose fractions. (**E**) Selected clusters from (D) enlarged. (**F**) Mean relative expression profiles across a single ATLAS-Seq gradient for clusters highlighted in (D), with corresponding Gene Ontology (GO) categories enriched within each cluster (right panel).

In this study, we applied ATLAS-Seq to mouse liver. Approximately 80% of mouse liver by weight is composed of hepatocytes (41), minimizing contributions from other cell types that could confound interpretation of fractionation profiles. In addition, previous studies of liver have performed velocity sedimentation, followed by fractionation and mass spectrometry, to generate a ‘fingerprint’ of co-fractionating proteins and protein complexes across the gradient (26). We performed similar velocity sedimentation of a detergent-free, post-nuclear liver lysate across a 10–50% sucrose gradient (Figure 1A). Notably, although velocity sedimentation only yields modest enrichment of particular organelles at specific densities relative to density equilibrium approaches, it can be advantageous for generating unique fingerprints for a variety of RNP, RNA and membrane-associated complexes across the full spectrum of the gradient. We collected 24 fractions from homogenized supernatant and subjected 17 with sufficient protein content (fractions 3–19) to mass spectrometry (Supplementary Table S1). The normalized abundance of known organelle markers including calnexin (endoplasmic reticulum, ER), clathrin (clathrin-coated vesicles), Gapdh (cytosol), Psma1 (proteasome) and catalase (peroxisome) were plotted across the gradient (Figure 1B) and showed patterns similar to previous studies (26). Importantly, we observed that these well-established organellar markers do not always peak at a specific density, but rather peak at different gradient fractions and exhibit distinct profiles, potentially reflecting the microenvironmental preferences of each protein in the cell.

Given our ability to separate proteins according to published expectations, we subsequently performed RNA-Seq on 22 out of 24 of the fractions with sufficient RNA content (fractions 3–24). Overall, gene expression profiles of fractions with similar densities were more highly correlated than fractions with greater density differences (Figure 1C). Two independent biological gradients were generated and analyzed, and similar profiles were observed across the transcriptome of the biological replicate gradient (Supplementary Figure S1, Supplementary Table S2). Given the high degree of concordance between transcriptome replicate gradients, we focused subsequent analyses on the gradient with a larger number of fractions, from which matched proteomic data were also generated. Unsupervised hierarchical clustering identified groups of RNAs among whose normalized expression profiles across the gradient were highly correlated (Figure 1D). 9269 genes were assigned to 635 distinct clusters; among these, 76 clusters contained at least 20 genes (Supplementary Table S3).

These clusters were subjected to Gene Ontology (GO) analysis. Of the 76 clusters, 53 showed enrichment for particular cellular compartments (cluster identities and GO results are in Supplementary Table S3). Similar to protein organelle markers, RNA profiles with strong GO enrichment did not always show a strong peak at any particular sucrose concentration; rather, profiles commonly showed modest enrichments of up to 4-fold at their greatest point (Figure 1D). For example, cluster 280 showed modest depletion in the center of the gradient and was highly enriched for categories including ‘chylomicron and plasma lipoprotein particle’. Clusters 522, 30 and 114 showed ∼2- to 3-fold enrichment at successively denser locations across the gradient, and revealed slightly different GO categories related to Golgi, aminoacyl-tRNA synthetase multienzyme complex, and mitochondrial respiration, respectively. Cluster 57, ∼2-fold enriched toward the denser part of the gradient, was enriched for proteasomal and mitochondrial categories (Figure 1E,F). Localization patterns of RNAs encoding proteasomal components have not been previously studied as a class and these results suggest that this subset of RNAs may exhibit a shared localization signature. Interestingly, the profiles of these RNAs are distinct from the peptide profile of a proteasomal marker, Psma1, as assessed by mass spectrometry. Overall, these observations show that RNAs with similar sedimentation properties often encode proteins known to co-associate or co-assemble in the cell.

### Sedimentation of RNA in ATLAS-Seq is influenced by factors beyond ribosome density

**Figure 2.**
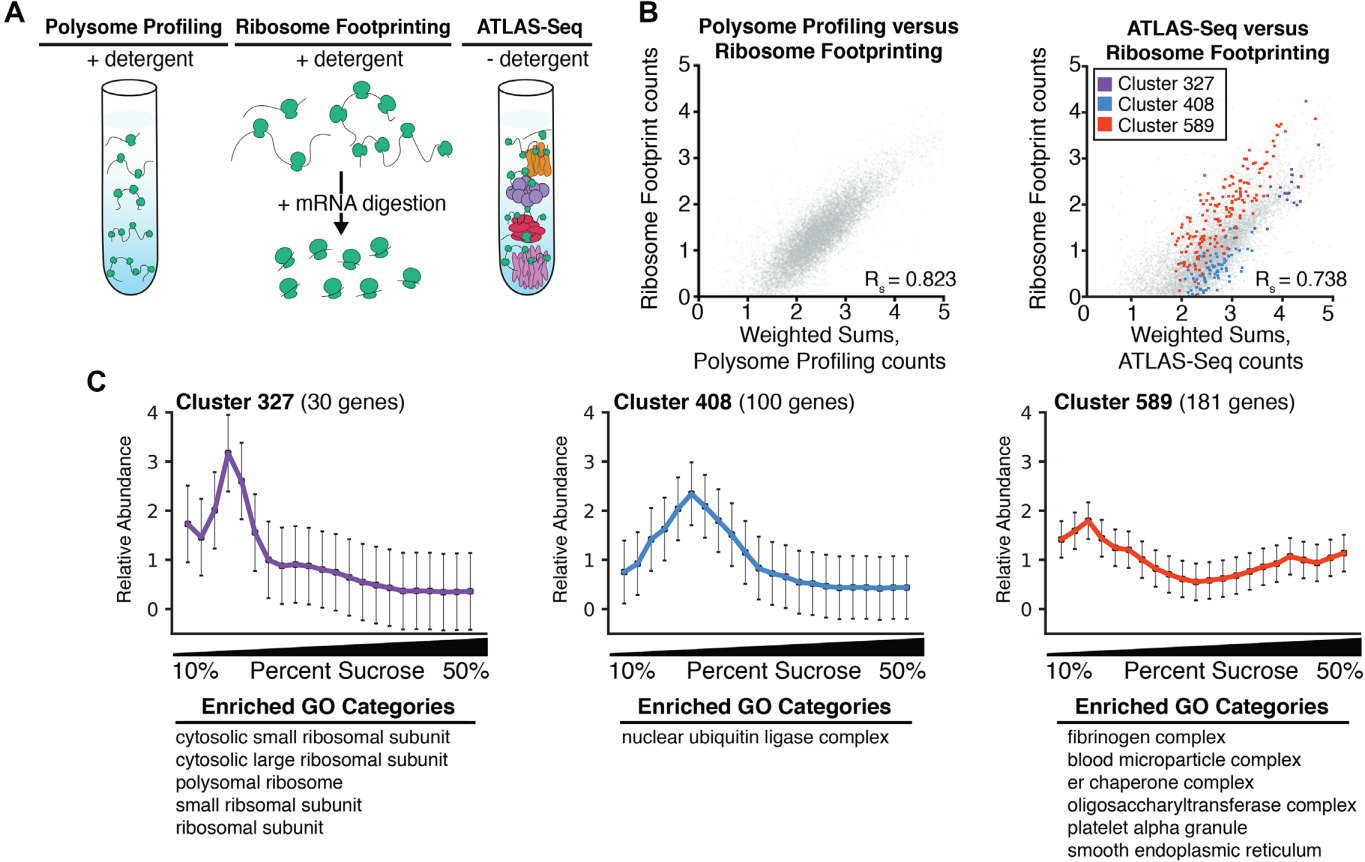
ATLAS-Seq profiles reflect a combination of subcellular microenvironment and ribosome occupancy. (**A**) Schematic illustrating sample preparation differences between polysome profiling, ribosome footprint profiling, and ATLAS-Seq. (**B**) Scatter plot of weighted sums of polysome profiling counts (see Methods) versus ribosome footprint profiling transcript per million (TPM) counts in HEK293T cells (top panel). Scatter plot of weighted sums of ATLAS-Seq counts (see Methods) and ribosome footprint profiling TPM counts in mouse liver (bottom panel). All genes are shown in gray, and genes in three clusters identified by hierarchical clustering of one ATLAS-Seq gradient (Figure 1D) are shown in red, purple, and blue. (**C**) Normalized ATLAS-Seq profiles for each of the three clusters highlighted in (B), along with GO categories for which they are enriched.

Both polysome profiling and ATLAS-Seq rely on separation by ultracentrifugation through a density gradient. However, an important difference is that polysome profiling employs detergents prior to loading onto the sucrose gradient to disrupt membranes and membranous organelles, whereas ATLAS-Seq does not (Figure 2A). To assess the extent to which ribosome density might influence ATLAS-Seq profiles, we analyzed published ribosome footprint profiles (42,43) and polysome profiles (44) together with our ATLAS-Seq profiles. As a first comparison, we correlated ribosome footprint counts to polysome profile counts (both from HEK293T cells) as estimated by a weighted sum of RNA across each polysome profile peak (see Materials and Methods). We observed a strong correlation (Pearson's *R* = 0.823), and importantly, a single cloud of points centered along a diagonal (Figure 2B, left panel) indicating that polysome profiling is mainly a measure of ribosome density rather than RNAs bound to larger cellular compartments or complexes as previously reported (37).

We then correlated ribosome footprint counts to ATLAS-Seq counts, also by weighted sums across each of the 22 fractions, mirroring calculations of polysome profiling counts above. A high correlation would imply that ATLAS-Seq mirrors a polysome gradient, and a low correlation would imply that ribosome occupancy cannot fully explain ATLAS-Seq profiles. We observed a weaker correlation (Pearson's *R* = 0.738) and also the presence of subsets of RNAs lying off the main diagonal (Figure 2B, right panel). Upon further inspection, we noticed that clusters we previously identified by hierarchical clustering (Figure 1D) separated away from the diagonal and were associated with specific GO categories (Figure 1E). For example, RNAs in cluster 589, a cluster predicted to be membrane-associated due to enrichment of RNAs encoding secreted proteins and ER components, appear less dense according to ATLAS-Seq relative to ribosome footprint profiling (Figure 2C). Overall, these observations suggest that the mild detergent-free homogenization conditions of ATLAS-Seq allow additional cellular components besides ribosomes to influence sedimentation, providing information about the cellular compartments with which RNAs are associated.

### ATLAS-Seq RNA localization patterns are consistent with those identified by orthogonal methods

**Figure 3.**
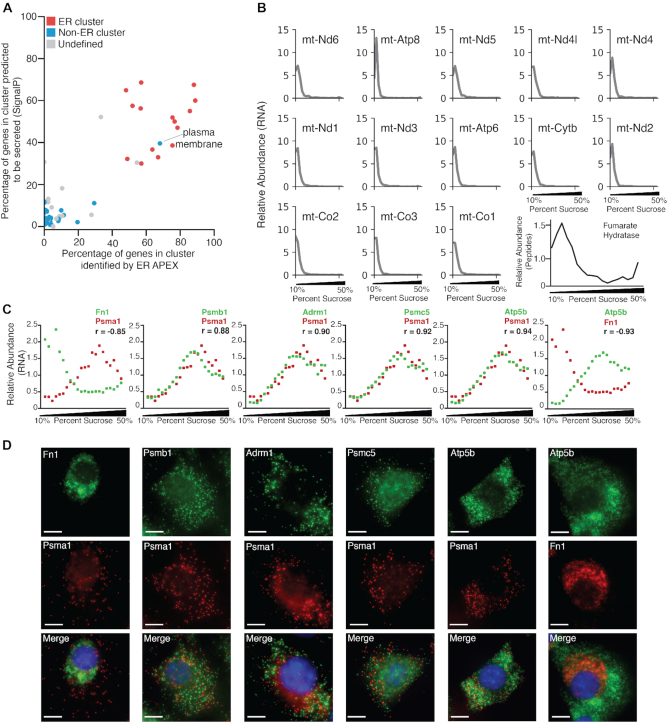
ATLAS-Seq reveals subcellular localization of RNAs in a manner consistent with other established techniques. (**A**) Gene clusters identified by ATLAS-Seq, plotted as a function of the proportion of genes within each cluster identified by ER APEX-RIP (x-axis) and the proportion of genes within each cluster predicted to be secreted by SignalP (y-axis). Clusters significantly enriched as determined by Fisher's exact test, in ER-related GO categories are shown in red, clusters with significant non-ER GO enrichment are shown in blue, and clusters with no significant GO enrichment are shown in gray. (**B**) Distribution of normalized TPMs across the ATLAS-Seq gradient for 13 genes identified to be mitochondrially-associated by APEX-RIP. Normalized mass spectrometry peptide counts for fumarate hydratase across the ATLAS-Seq gradient are shown in black. Pearson correlation coefficients between TPMs for each RNA and fumarate hydratase peptide counts are shown. (**C**) Normalized TPM profiles across one ATLAS-Seq gradient for RNAs encoding fibronectin 1 (Fn1), proteasomal subunit A1 (Psma1), proteasomal subunit B1 (Psmb1), Proteasomal ubiquitin receptor (Adrm1), 26s proteasome regulatory subunit 8 (Psmc5), and ATP synthase subunit beta (Atp5b) in green or red as labeled. Pearson correlation coefficients between each pair of RNAs are also listed. (**D**) smiFISH for RNAs encoding Fn1, Psma1, and Psmb1, Adrm1, Psmc5, Atp5b in NIH 3T3 cells. Fn1 exhibits a perinuclear pattern, whereas Psma1 and Psmb1 are distributed throughout the cytoplasm. Nuclei were stained by DAPI (blue), and the same scale bar applies to all images (10 μm).

Although our analyses thus far suggested that ATLAS-Seq can reveal information about the subcellular location of RNAs, we sought to compare these predictions to observations made using orthogonal methods. Crosslinking of RNAs to proteins labeled by APEX has been used to capture RNAs localized to specific subcellular locations, for example the outer surface of the ER or the mitochondrial matrix (45). First, we analyzed RNAs published to be associated with the ER according to APEX-RIP. For each ATLAS-Seq cluster, we computed the fraction of RNAs determined to be ER-associated by APEX-RIP as well as the fraction of RNAs predicted to have a signal sequence according to SignalP (46). We plotted a scatter of these metrics for each cluster (Figure 3A) and highlighted each cluster in red if it was significantly enriched for any ER-related GO categories (Supplementary Table S3) Clusters identified to be ER-associated by ATLAS-Seq showed enrichment for RNAs identified by ER APEX-RIP and RNAs with high SignalP scores. Although one ATLAS-Seq cluster did not show ER-related GO enrichment, it did show enrichment for ‘plasma membrane’, and proteins in the plasma membrane are typically derived from proteins synthesized, processed, and trafficked via the endomembrane system (47,48). Interestingly, although all of these clusters were enriched for ER-related GO terms, some exhibited distinct profiles that could be further stratified by specific GO subcategories. (Supplementary Figure S2). For example, cluster 598 was enriched for the ER chaperone complex, whereas cluster 280 was enriched for RNAs encoding proteins found in lipoprotein microparticles. Therefore, our approach may provide finer resolution to identify subclusters corresponding to distinct ER microenvironments.

To further assess whether our gradient could reveal co-localized RNAs, we analyzed the 13 protein-coding mRNAs of the mitochondrial genome, which are known to reside in the mitochondria. Profiles of these RNAs highly correlated with each other and also with the mass spectrometry profile of a mitochondrial resident protein, fumarate hydratase (Figure 3B). The high concordance of these profiles suggests that our approach preserves the association between RNAs inside the mitochondria and proteins associated with the organelle. Taken together, these analyses confirm that ATLAS-Seq yields information related to the subcellular localization of RNA species, and that profiles of RNAs with unknown localization patterns may be used to predict their local microenvironment.

We then sought to explore subcellular distributions of RNAs for which little is known. Eleven out of 19 RNAs encoding the proteasome core complex were found in ATLAS-Seq clusters 53 and 57. The localization of the proteasome itself is well studied and has been observed to play a key role in mitochondrial biogenesis (49). Interestingly, both proteasomal clusters, 53 and 57, also contained a number of nuclear-encoded mitochondrial RNAs (Supplementary Table S3).

To assess whether results from our ATLAS-Seq analysis were consistent with an imaging-based approach, we performed single-molecule inexpensive FISH (smiFISH) on 4 proteasomal core complex RNAs (Psma1, Psmb1, Psmc5 and Adrm1), a nuclear-encoded mitochondrial RNA (Atp5b), and a signal sequence-containing RNA (Fn1). All proteasome-encoding RNAs and Atp5b exhibited similar ATLAS-Seq profiles, whereas Fn1 exhibited a highly distinct profile that was representative of RNAs encoding secreted proteins (Figure 3C). SmiFISH for Fn1 RNA in both liver (Supplementary Figure S3) and adherent NIH 3T3 cells (Figure 3D) revealed a perinuclear pattern, consistent with the presence of a signal sequence and localization to the ER. Psma1, Psmb1, Psmc5, Adrm1 and Atp5b were found throughout the cytoplasm in a pattern distinct from that of Fn1. Interestingly, in spite of highly overlapping proteasomal and mitochondrial ATLAS-Seq profiles, smiFISH did not reveal strong spatial co-localization. This indicates that while ATLAS-Seq cannot provide information about the precise spatial location of an RNA, it may rather provide information about local microenvironments within a particular subcellular region—a property that is often difficult to discern by image-based methods.

### Comparing sedimentation patterns of RNAs and the proteins they encode

**Figure 4.**
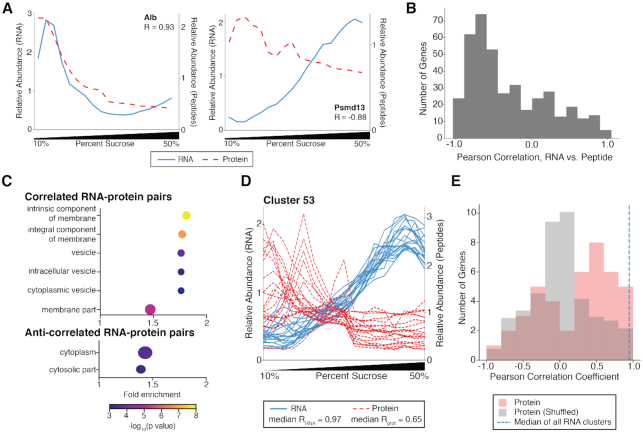
Most RNAs are anti-correlated with the proteins they encode in the ATLAS-Seq gradient. (**A**) Normalized TPM (blue line) and peptide counts (red line) across the ATLAS-Seq gradient for Albumin (Alb, top panel), and 26S proteasome non-ATPase regulatory subunit 2 (Psmd2, bottom panel). Pearson correlation coefficients between RNA and protein are shown. (**B**) Distribution of Pearson correlation coefficients between RNAs and the proteins they encode across the ATLAS-Seq gradient for 404 genes. (**C**) Cellular compartment GO categories enriched in genes whose RNAs are strongly correlated with the proteins they encode. The size of each dot is determined by the number of genes (also listed next to point) found in that GO category. Fold enrichment was calculated by the observed number of genes in a GO category divided by the expected number of genes in that category (see Methods) (top panel). Cellular compartment GO categories enriched in genes whose RNAs strongly anti-correlate with the proteins they encode (bottom panel). (**D**) Normalized TPM (blue lines) and peptide counts (red lines) across the ATLAS-Seq gradient for genes with both ATLAS-Seq and mass spectrometry data in Cluster 53, which is enriched for proteasome genes. The median pairwise correlation among all RNAs and among all proteins in the cluster are listed. (**E**) Histogram of median pairwise correlations of protein profiles (red) for all clusters containing at least two proteins. Median pairwise correlations were also computed using shuffled RNA-protein assignments and plotted (gray). For reference, the median of all median pairwise RNA correlations across all RNA clusters is indicated in blue dashed line.

A long-standing question is the extent to which RNAs co-localize with the proteins they encode. It is well established in neurons that many synaptically localized RNAs encode locally translated proteins, and therefore show co-localization (10). In contrast, in the mouse intestinal epithelium, localization of many mRNAs is distinct from their encoded proteins (50). Although ATLAS-Seq cannot truly assess co-localization of RNAs and proteins in space, it can assess the extent to which they co-sediment. We compared normalized protein profiles to normalized RNA profiles across the sucrose gradient, limiting these analyses to genes for which we had both reasonable RNA-Seq read coverage and mass spectrometry peptide counts (404 genes in total, Supplementary Table S5). As examples, we show that RNA and protein profiles for Alb (albumin) were highly concordant (Pearson's *R* = 0.93), whereas the RNA and protein for Psmd13, a 26S proteasome subunit protein, were anti-correlated (Pearson's *R* = −0.88) (Figure 4A). We plotted a histogram of these correlations across all genes for which we could obtain reproducible RNA and protein data and observed that most genes exhibited a negative correlation; that is, RNA and protein exhibited anti-correlated sedimentation profiles (Figure 4B). This suggests that in liver, the majority of RNAs are not localized to the same subcellular region as the steady state destination of their protein counterparts or are in a microenvironment distinct from the protein they encode. GO analysis revealed that genes with a high correlation between their RNA and protein counterparts were enriched for secretion and/or endomembrane-trafficking, whereas highly anti-correlated genes were enriched for cytosolic genes (Figure 4C, Supplementary Table S5). Indeed, the most positively correlated genes (top 20th percentile) contained signal sequences ∼38% of the time, consistent with their translation at the ER membrane, whereas the most negatively correlated genes (bottom 20th percentile) contained signal sequences only ∼14% of time (Supplementary Figure S4C). Thus, although proteins of the ER colocalize with their RNA, most RNAs and their encoded proteins did not co-sediment in this context.

Although most RNAs do not co-sediment with the proteins they encode, our previous Gene Ontology analysis of RNA clusters (Figure 1) suggested that proteins encoded by co-sedimenting RNAs act in similar biological pathways or cellular compartments. We therefore grouped proteins by their RNA cluster assignments and analyzed their sedimentation patterns. For example, RNAs in Cluster 53, enriched for the proteasome complex, showed enrichment toward the bottom of the gradient and were highly correlated with one another, showing a median correlation among all pairwise comparisons of 0.97 (Figure 4D). Interestingly, the proteins encoded by these RNAs also tended to correlate with one another, showing a median pairwise correlation of 0.65. To assess this globally, we analyzed every cluster for which there were at least two proteins assessed by mass spectrometry and obtained the median pairwise correlation among all proteins in each cluster. These median correlation values were enriched for positive values (Figure 4E, pink bars), and were much greater than when computed using shuffled RNA–protein assignments (Figure 4E, gray bars). This analysis provides further evidence that the sedimentation patterns of RNAs contain information about the subcellular localization of the proteins they encode.

### Alternative isoforms are differentially localized across the ATLAS-Seq gradient

**Figure 5.**
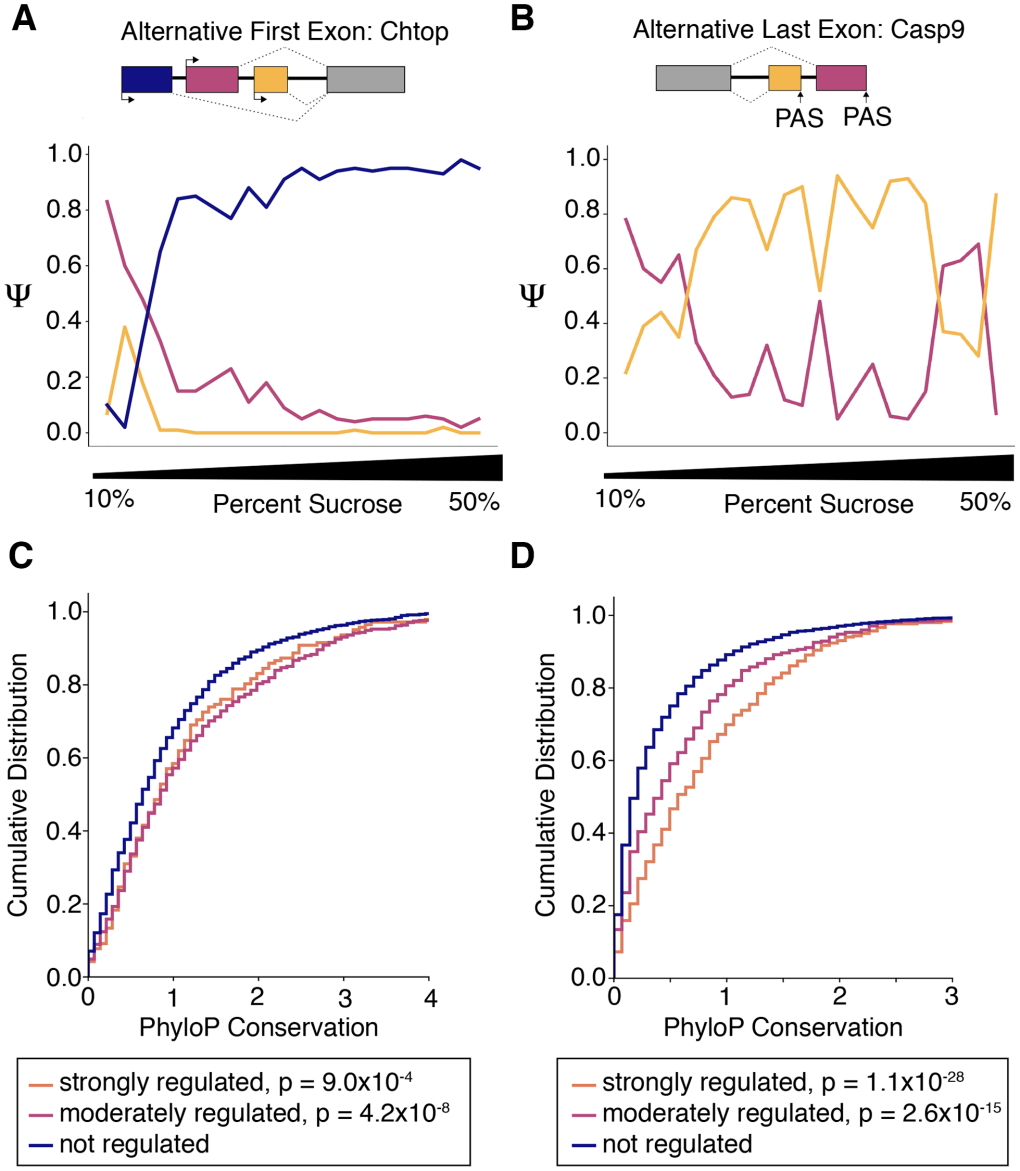
Alternative first and last exons exhibit differential profiles across the ATLAS-Seq gradient. (**A**) Ψ values across one ATLAS-Seq gradient for AFE isoforms of Chromatin target of PRMT1 protein, Chtop. (**B**) Ψ values across one ATLAS-Seq gradient for ALE isoforms of Caspase-9, Casp9. (**C**) Cumulative distribution of PhyloP conservation scores for AFE isoforms, separated by strongly regulated (ΔΨ > 0.5), moderately regulated (0.5 < ΔΨ > 0.25), and non-regulated (ΔΨ < 0.25) isoforms. P values were determined by Wilcoxon rank-sum test, comparing each regulated group to the non-regulated group. (**D**) Cumulative distribution of PhyloP conservation scores for ALE isoforms, similar to (C).

We next investigated whether alternative isoforms from the same gene loci exhibit differential sedimentation patterns across the ATLAS-Seq gradient. Because untranslated regions have known roles in regulating RNA localization, we focused on alternative UTR isoforms for these studies. We considered both alternative first exons (AFE, generated by alternative promoter usage and splicing to a constitutive exon) and alternative last exons (ALE, generated by alternative splicing and/or polyadenylation). We quantitated the proportion of each isoform present in each fraction of the gradient, labeled percent spliced in, or PSI (Ψ) (Supplementary Table S6). After limiting analyses to isoforms for which Ψ could be confidently estimated (see Materials and Methods), we found 152 AFEs and 332 ALEs for which the maximum difference in Ψ (ΔΨ) across the gradient for any pair of isoforms was >0.5. For example, one AFE isoform for Chtop showed a ΔΨ of 0.96 towards the densest part of the gradient (Figure 5A). Similarly, one ALE isoform of DNA–Caspase-9 (Casp9) showed a ΔΨ of 0.73 (Figure 5B) towards the densest part of the gradient. These observations confirm distinct subcellular distributions of alternative isoforms, as revealed by sucrose density fractionation.

If the relative abundance of these alternative isoforms is important for cell function, they may contain sequences subject to positive selection through evolution. To determine whether isoforms with differential sedimentation patterns are more phylogenetically conserved, we measured their conservation using PhyloP scores. AFE isoforms showing strong (ΔΨ > 0.5) and moderate (0.5 < ΔΨ < 0.25) differential sedimentation showed similar conservation scores but were more highly conserved than isoforms lacking differential localization (ΔΨ < 0.25) (Figure 5C). In contrast, the extent of differential localization of ALE isoforms correlated with conservation across all three groups, e.g. more strongly localized ALE isoforms were more highly conserved suggesting their enrichment for functional features.

### ATLAS-Seq profiles of RBPs correlate with target mRNAs

RBPs can control the RNA localization of their targets, but few RBP-RNA pairs have been functionally validated in this context. Because each RNA interacts with many RBPs, the impact of each RBP-RNA interaction may only subtly influence final destination of that RNP. Therefore, analysis of many RNAs showing similar sedimentation patterns may be required to provide the power necessary to identify potentially weak, yet true, significant signals.

**Figure 6.**
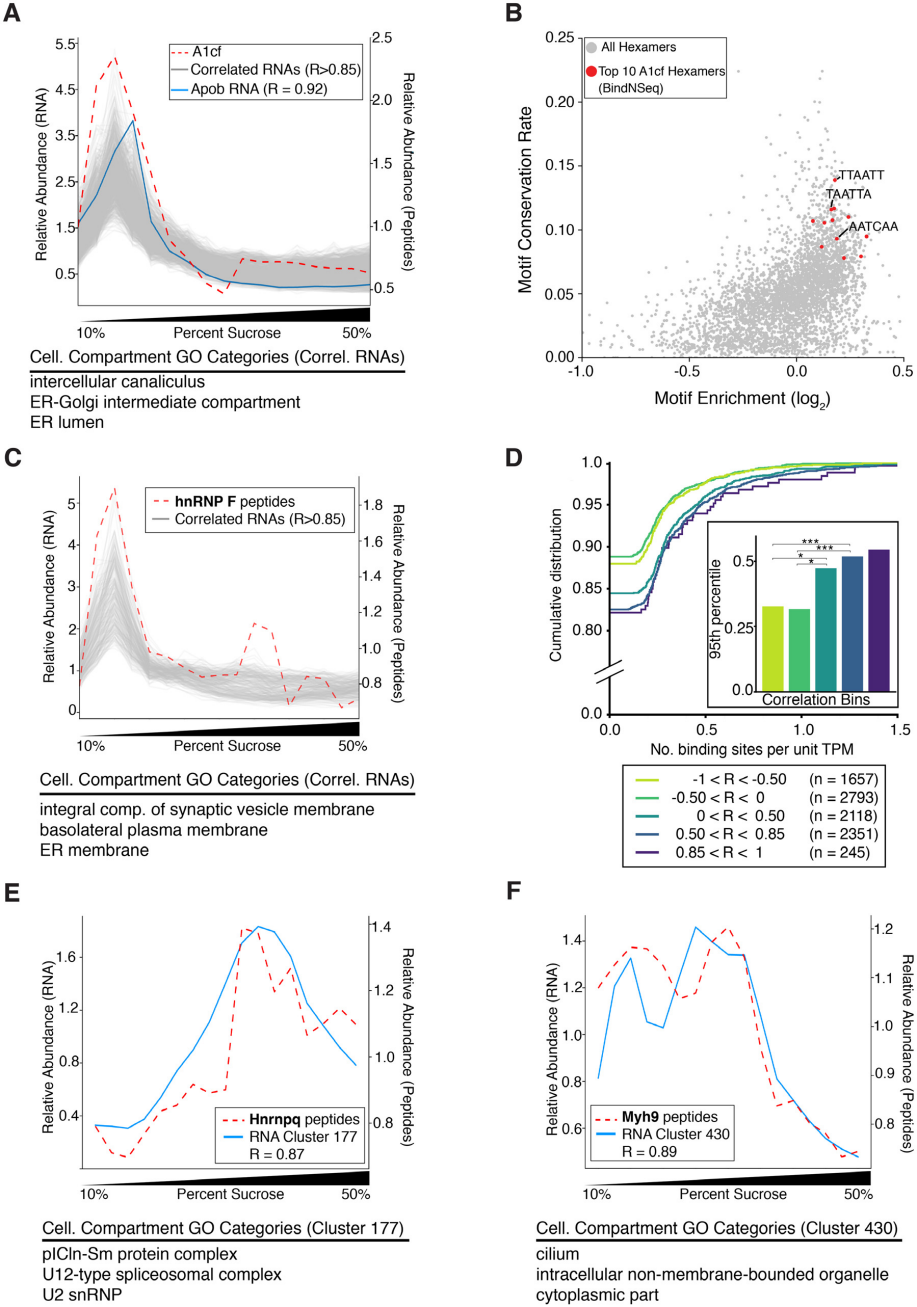
ATLAS-Seq reveals associations between RNA binding proteins and their RNA targets. (**A**) Distribution across the ATLAS-Seq gradient of relative peptide counts of APOBEC1 Complementation Factor (A1cf, red dashes), and normalized TPMs for apolipoprotein B (Apob, blue line). Pearson's R between A1cf and Apob = 0.92. Also shown are normalized TPMs for 894 RNAs correlating with a Pearson's *R* > 0.85 (gray). Shown below are GO Cellular Compartment terms associated with these RNAs. (**B**) Hexamers plotted by log_2_(foreground / background counts) on the x-axis and conservation rate on the y-axis, where foreground counts were obtained from 3′ UTRs in the set of 894 RNAs correlating > 0.85 from (A) and background counts were obtained from all other RNAs in the gradient. Conservation rate was computed across all mouse Refseq 3′ UTRs as fraction of instances showing full conservation across mouse, human, rat, and dog multi-alignments. The top 10 BindNSeq A1cf hexamers are highlighted in red. (**C**) Relative peptide counts for heterogeneous nuclear ribonucleoprotein F (hnRNP F, red dashes) and normalized TPMs for RNAs correlating with Pearson's *R* > 0.85 (gray). (**D**) Cumulative distribution of the number of hnRNP F CLIP binding sites per unit TPM for groups of RNAs separated by Pearson's correlation to relative abundance of hnRNP F peptides. *P < 0.05, **P < 0.001 as assessed by Wilcoxon rank-sum test. (**E**) Relative peptide counts for heterogeneous nuclear ribonucleoprotein Q (hnRNP Q/Syncrip, red dashes) and mean TPM profile for the RNA cluster best correlating with hnRNP Q peptide counts (blue line). Shown below are GO Cellular Compartment terms enriched in that cluster. (**F**) Relative peptide counts for Myosin-9 (Myh9, red dashes) and mean TPM profile for the RNA cluster best correlating with Myh9 peptide counts (blue). Shown below are GO Cellular Compartment terms enriched in that cluster.

To test the hypothesis that RBPs and their RNA targets might co-sediment through the gradient, we first analyzed a known example of an RBP-RNA pair. The RNA binding protein APOBEC1 complementation factor, A1cf, is known to bind and edit the RNA encoding Apolipoprotein B (Apob) (51). The relative abundance of A1cf peptides and Apob RNA were strongly correlated across the gradient (Pearson's *R* = 0.92, Figure 6A). Given this correlation, we hypothesized that additional RNAs whose profiles strongly correlated with A1cf might also be binding partners of A1cf. We identified 894 RNAs whose profiles correlated strongly with A1cf (Pearson's *R* > 0.85); these RNAs encoded proteins enriched for GO Cellular Compartment categories such as ER, golgi, endosome, and vesicles (Figure 6A). Enriched GO Biological Processes included lipid localization/transport and the Endoplasmic Reticulum-associated protein degradation (ERAD) pathway – functions known or proposed to be associated with A1cf (52). Similar results were observed in a separate replicate gradient (Supplementary Figure S5). Notably, binding motifs for A1cf identified *in vitro* by BindNSeq were enriched in the 3′ UTRs of these 894 RNAs relative to all other RNAs in the gradient; these hexamers were also more highly conserved than other hexamers in all 3′ UTRs of mouse mRNAs (Figure 6B).

To further assess whether the abundance of specific RBPs across the gradient might be associated with the localization of their target RNAs, we identified RBPs in our mass spectrometry dataset for which functional binding data was also publicly available. We focused on hnRNP F, for which there is publicly available CLIP-Seq data from HEK293T cells (53). We correlated all RNAs in our gradient to the peptide profile for hnRNPF and separated them by Pearson's correlation coefficient. The most strongly correlating RNAs (Pearson's *R* > 0.85) were enriched for specific GO categories, including ER membrane (Figure 6C, Supplementary Figure S5). We analyzed mouse orthologs of human RNAs bound by hnRNP F according to CLIP and found that more highly correlated RNAs showed a greater density of CLIP binding in 3′ UTRs relative to less correlated or anti-correlated RNAs, as measured by number of binding sites per unit of gene expression (Figure 6D).

To uncover additional RBP-RNA relationships that may drive co-sedimentation patterns, we identified 134 RBPs (see Methods) supported by mass spectrometry peptides across our ATLAS-Seq gradient. We correlated their profiles to all ATLAS-Seq RNA clusters (Supplementary Figure S6) and found 71 RBPs whose peptide counts correlated to our previously defined RNA cluster profiles (Pearson's R>0.85). While functional connections between most of these RBP-RNA cluster pairs are unknown, some relationships observed are consistent with known functions of the RBPs. For example, heterogeneous nuclear ribonucleoprotein Q (hnRNP Q/SYNCRIP) correlated most strongly with cluster 177 (Pearson's *R* = 0.86), which contains RNAs encoding proteins in the pICln-Sm protein complex, the U12-type spliceosomal complex, and U2snRNPs (Figure 6E, Supplementary Table S7). Consistent with this pairing, HnRNP Q interacts with Survival of Motor Neuron (SMN) complex (54), is a component of the spliceosome, and has been proposed to link the SMN complex to splicing functions (55). As another example, we observed that Myosin-9 (Myh9) correlates best with cluster 430 (*R* = 0.90), which contains RNAs encoding cilium components (Figure 6F, Supplementary Table S7). Myh9 is an RBP that also contains a motor domain and has been shown to compete with Myh10 to inhibit cilium biogenesis (56). Taken together, these results support the ability of ATLAS-Seq to predict RBP-RNA associations and their regulatory connections.

## DISCUSSION

We have used ATLAS-Seq to uncover unexpected relationships between sucrose gradient sedimentation profiles of RNAs encoding proteins involved in similar biological functions. Deep sequencing of RNA transcriptome-wide and mass spectrometry of peptides with high resolution across the gradient facilitated the discovery of these relationships and characterized the presence of cellular microenvironments to which RNAs are sorted. Surprisingly, subtle differences in profile shape can resolve differences in the composition of cellular compartments. These profiles likely reflect not only engagement with large macromolecules such as the ribosome, but also membranes and other structures with distinct physiochemical properties. We observed that these interactions were reflected in the divergence of ribosome footprint profiles from ATLAS-Seq. Future studies directly comparing polysome profiles to ATLAS-Seq or other gradients prepared by diverse detergents, cytoskeletal disruptors, or other agents might further elucidate how various interactions drive sedimentation profiles.

Distinct microenvironments in the cell arising from these interactions—the sum of weak attractive and repulsive forces between biomolecules—may create the appropriate settings for translation, sorting, decay, and other cellular processes. Although these specialized environments are sometimes membrane-bound organelles, our observations suggest they may also reflect membrane-less organelles in the cytoplasm such as the proteasome, sites of spliceosome component assembly, or even RNP granules. These RNP granules could contain single mRNAs bound to multiple RBPs, or perhaps supra-molecular assemblies in which multiple RNPs are linked via protein–protein, protein–RNA or even RNA–RNA interactions. Thus, there exists spatial organization among thousands of RNAs revealed by physical separation across a density gradient. The observations here provide a blueprint for how RNAs might map to specific subcellular microenvironments in liver cells and provide insights into higher scale organization of the transcriptome.

Interestingly, correlations of RNA to their encoded proteins revealed that most RNA-protein counterparts are not co-localized, but that some are, most notably those encoding membrane and secreted proteins. In these cases, the co-localization may reflect co-translational insertion into specific lumenal compartments. However, both RNAs with and without signal peptide sequences often co-sedimented, suggesting that there may be additional signals within RNA that influence their localization. Notably, proteins encoded by co-sedimenting RNAs also tend to co-sediment, suggesting regulatory mechanisms that bridge the subcellular localization of each molecule. This has been previously observed for specific mRNAs at the isoform level; for example, localization of some proteins has been shown to be directly influenced by 3′ UTRs of their mRNAs via association with membraneless organelles such as TIGER domains (57). This is consistent with our findings that isoforms with distinct last exons and 3′ UTRs showed distinct sedimentation patterns associated with increased phylogenetic conservation. Indeed, alternative 3′ UTRs have been shown to localize mRNAs to neurites versus soma (15). Whether RNAs co-localize with their encoded proteins may also depend on cell type and/or cell state and remains to be further characterized.

A key goal in studies of RNA sorting and localization is to identify RNA elements and RBPs that might define subcellular localization of RNAs and locally translated proteins. Only a small fraction of putative RBPs have been functionally characterized, but co-sedimentation of RBPs and RNA targets may reveal functional interactions. In summary, high resolution subcellular fractionation on a transcriptome-wide scale can provide important insights into the regulation of higher order, subcellular compartmentalization of mRNAs by revealing groups of RNAs that co-segregate within the cell and implicating post-transcriptional processes and *trans*-factors associated with these microenvironments.

## DATA AVAILABILITY

Raw sequencing reads for all samples are available through the NCBI via GEO Accession GSE140630. An interactive browser that allows users to explore profiles of transcripts, proteins, and ATLAS-Seq clusters can be found at http://ericwanglab.com/atlas.php.

## Supplementary Material

gkaa334_Supplemental_Files
