# Supplementary material for: Transcriptome-wide organization of subcellular microenvironments revealed by ATLAS-Seq
Source: Nucleic Acids Res. 2020 May 18;48(11):5859–72. doi: 10.1093/nar/gkaa334 (PMC7293051; doi:10.1093/nar/gkaa334)
Supplement: gkaa334_Supplemental_Files [file gkaa334_supplemental_files.zip › Adekunle_NAR_supplement_final.pdf]

Supplemental Information

Supplemental information includes 5 figures and 9 tables and can be found below.

Supplemental Figure Legends

Figure S1. Related to Figure 1. Correlation in gene expression between ATLAS-Seq gradient replicates.
Heatmap showing Pearson correlation coefficients of gene expression between all pairs of RNA-Seq libraries from sucrose fractions from both ATLAS-Seq gradients. Gradient 2 is shown in the main **Figure 1**.

Table S1. Related to Figure 1.
Mass spectrometry counts for all proteins identified in this study. Gene names are listed, followed by Uniprot protein accession IDs, a description of the protein, and relative peptide abundances in each fraction for the protein. Mass spectrometry was conducted on gradient 2, with 1 technical replicate. The data reflect the aggregate of both technical replicates.

Table S2. Related to Figure 1.
TPM values for all Refseq Genes for which at least 1 fraction has TPM > 1. Data from both gradients are shown here, and the gradient number is indicated in the worksheet name. Data from gradient 2 is highlighted in all main figures.

Table S3. Related to Figure 1.
Worksheet 1: GO enrichments for each RNA cluster. Cluster number is in the left-most column, followed by GO enrichment. Subsequent columns show the observed number of genes in the GO category, the expected number of genes in the GO category, the fold enrichment, and p-value. The final column marks whether a cluster is designated as an ER cluster as defined by GO analysis.
Worksheet 2: Hierarchical clustering assignments from clustering of gradient 2.
Worksheet 3: Clusters analyzed in Figure 3A (those that contain more than 20 genes).

Figure S2. Related to Figure 3. ER clusters possess distinct differences in subgroups of ER organelles they encode. A) Heatmap of GO enrichment categories for different ER clusters highlighted in Fig. 3A. B) Normalized TPM profiles across the gradient for ER clusters identified in Figure 3A.

Figure S3. Related to Figure 3. smFISH of mouse liver reveals subcellular localization of RNAs in a manner consistent with other established techniques. smiFISH for RNAs encoding Fn1, Psma1, and Psmb1, Adrm1 in 7 μM cryosections of FVB mouse liver. Nuclei were stained by DAPI (blue).

Table S4. Related to Figure 3.

Probe sequences for smiFISH analyses. Gene names and probe sequences are provided for each smiFISH probe set. For the smiFISH, Y-flap was used as the secondary probe in all cases with either Cy3 or Cy5 used as the fluorophore indicated.

Figure S4. Related to Figure 4. Most RNAs are anti-correlated with the proteins they encode across the ATLAS-Seq gradient. A) Normalized TPM (red line) and peptide counts (blue line) across the ATLAS-Seq replicate gradient 1 for Albumin (Alb, top panel), and 26S proteasome non-ATPase regulatory subunit 2 (Psmd2, bottom panel). Pearson correlations between RNA and protein are shown. B) Distribution of Pearson correlations between RNAs from gradient 1 and the proteins they encode (from gradient 2) for 404 genes. Dashed lines denote correlation thresholds used in subsequent GO analyses. C) Proportion of genes from **Figure 4B** predicted to be secreted by SignalP. Genes were separated into two groups – the top n^{th} percentile and the bottom n^{th} percentile. The proportion of each group containing genes with signal sequences is shown. Top n^{th} percentiles are depicted in light gray and bottom n^{th} percentiles are depicted in dark gray.

Table S5. Related to Figure 4.
Worksheet 1: RefSeq gene symbol followed by the Pearson's correlation coefficient between the RNA and protein of each gene, sorted by most to least correlated.
Worksheet 2: GO enrichment results (by Gorilla GO analysis package) sorted from most to least correlated. Highest ranked values are for genes whose RNA and protein counterparts are the most positively correlated.
Worksheet 3: GO enrichment results for genes whose RNA and protein counterparts are the most negatively correlated.

Table S6. Related to Figure 5.
Worksheet 1. RefSeq gene symbol followed by AFE psi values for each fraction of gradient 2.
Worksheet 2. RefSeq gene symbol followed by AFE psi values for each fraction of gradient 1.
Worksheet 3. RefSeq gene symbol followed by ALE psi values for each fraction of gradient 2.
Worksheet 4. RefSeq gene symbol followed by ALE psi values for each fraction of gradient 1.

Figure S5. Related to Figure 6. Selected RBPs and co-sedimenting RNAs in ATLAS-Seq gradient 1. A) Normalized mass spectrometry peptide counts for heterogeneous nuclear ribonucleoprotein F (dashed red, hnRNP F) and mean normalized TPMs for RNAs that correlate with a Pearson correlation coefficient > 0.85 (gray). Top GO cellular compartment enrichment categories are listed below the figure. B) Normalized mass spectrometry peptide counts for APOBEC1 Complementation Factor (dashed red, A1CF) and mean normalized TPMs for RNAs that correlate with a Pearson's correlation coefficient greater than 0.85 (gray). Shown below each panel are cellular compartment GO categories for the best correlating RNAs.

Figure S6. Related to Figure 6. Correlations of RBP peptide profiles to ATLAS-Seq profiles. Heatmap of Pearson's correlation between each RBP peptide (from mass spectrometry) to each ATLAS-Seq cluster profile containing at least 20 genes. Both RBPs and ATLAS-Seq profiles were hierarchically clustered. The cluster dendrogram is shown above.

Table S7. Related to Figure 6.
Worksheet 1. RefSeq gene symbol for each RBP followed by most correlated cluster and the Pearson's correlation coefficient between the peptide profile for the RBP profile and the mean profile of the RNA cluster.
Worksheet 2. RefSeq gene symbol for each RBP along with the top 5 most correlated clusters. Pearson's R values are provided.

Figure S1

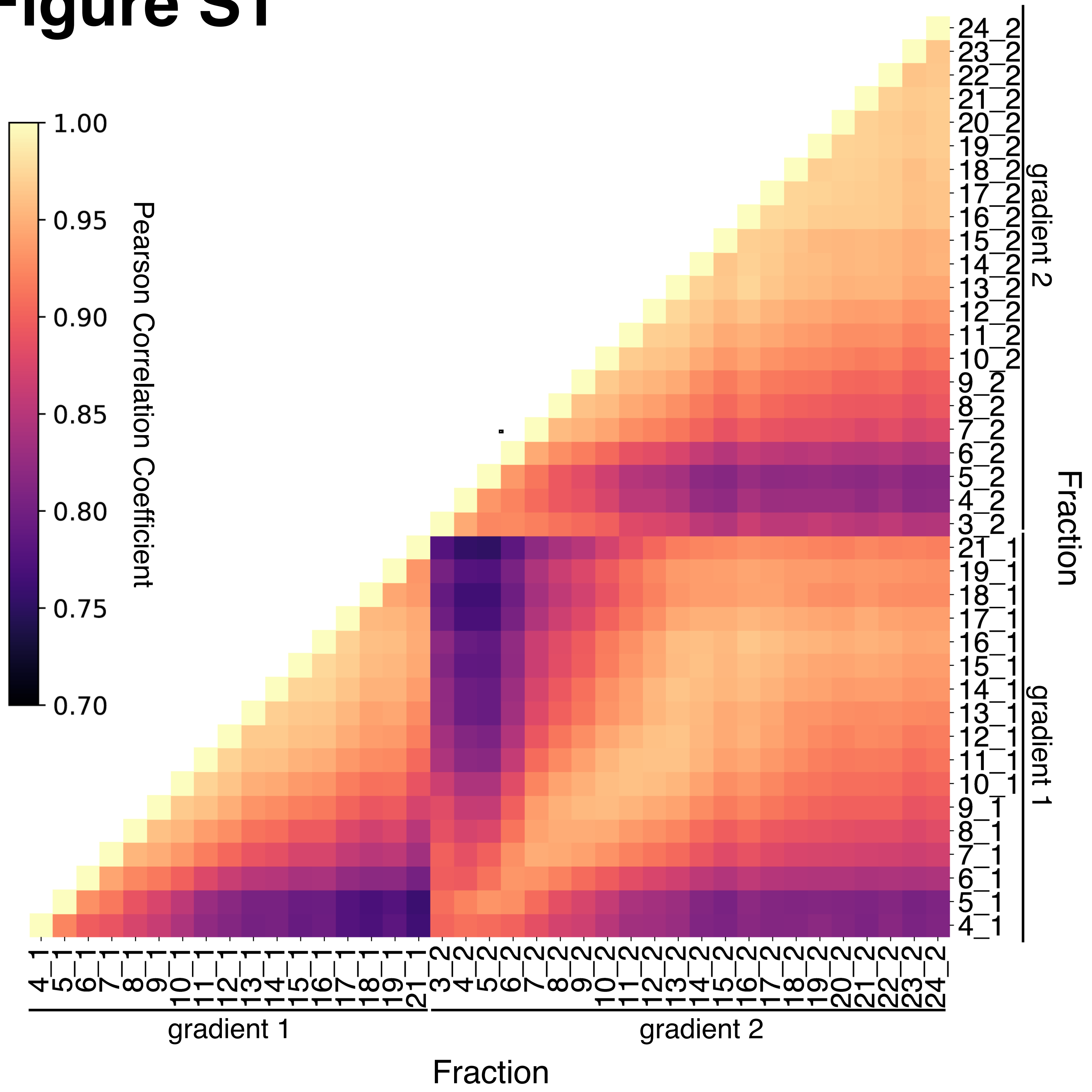

1.00
0.95
0.90
0.85
0.80
0.75
0.70
Pearson Correlation Coefficient
24_2
23_2
22_2
21_2
20_2
19_2
18_2
17_2
16_2
15_2
14_2
13_2
12_2
11_2
10_2
9_2
8_2
7_2
6_2
5_2
4_2
3_2
21_1
19_1
18_1
17_1
16_1
15_1
14_1
13_1
12_1
11_1
10_1
9_1
8_1
7_1
6_1
5_1
4_1
gradient 2
gradient 1
Fraction
4_1
5_1
6_1
7_1
8_1
9_1
10_1
11_1
12_1
13_1
14_1
15_1
16_1
17_1
18_1
19_1
21_1
3_2
4_2
5_2
6_2
7_2
8_2
9_2
10_2
11_2
12_2
13_2
14_2
15_2
16_2
17_2
18_2
19_2
20_2
21_2
22_2
23_2
24_2
gradient 1
gradient 2
Fraction

Figure S2

A

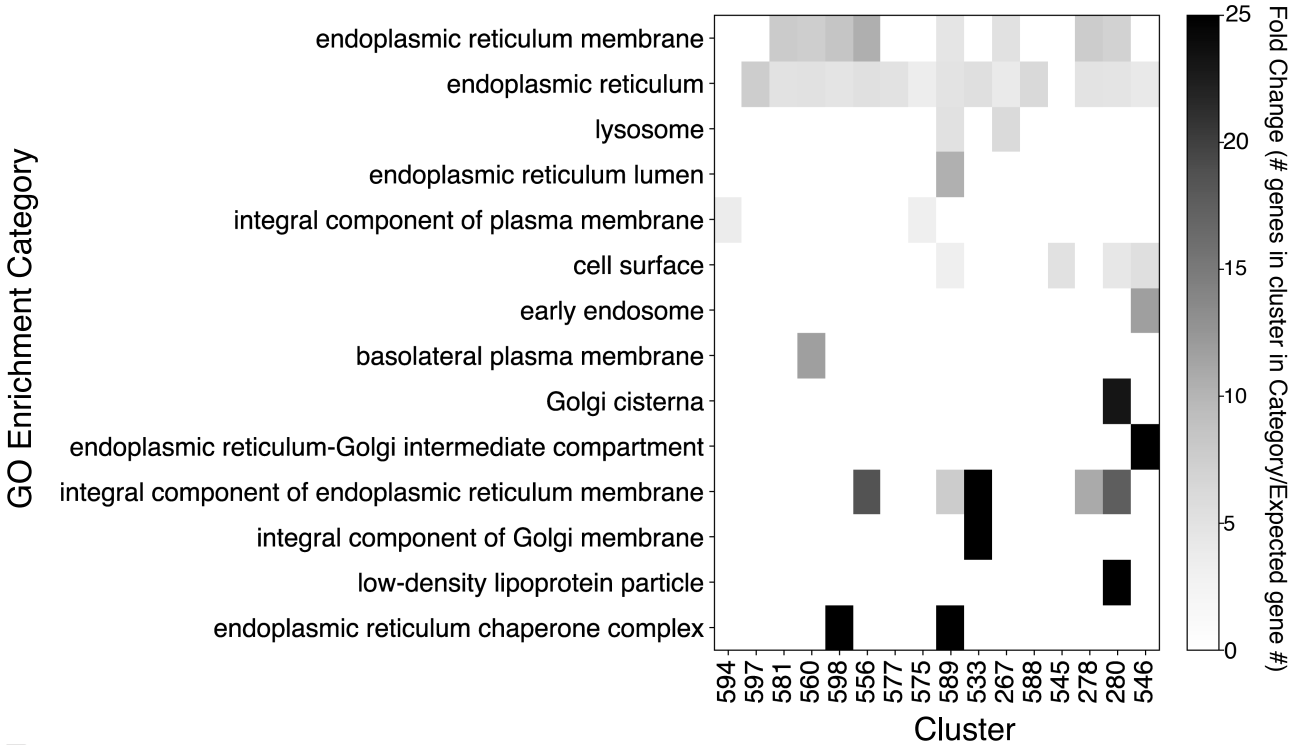

endoplasmic reticulum membrane
endoplasmic reticulum
lysosome
endoplasmic reticulum lumen
integral component of plasma membrane
cell surface
early endosome
basolateral plasma membrane
Golgi cisterna
endoplasmic reticulum-Golgi intermediate compartment
integral component of endoplasmic reticulum membrane
integral component of Golgi membrane
low-density lipoprotein particle
endoplasmic reticulum chaperone complex
GO Enrichment Category
594 597 581 560 598 556 577 575 589 533 267 588 545 278 280 546
Cluster
0 5 10 15 20 25
Fold Change (# genes in cluster in Category/Expected gene #)

B

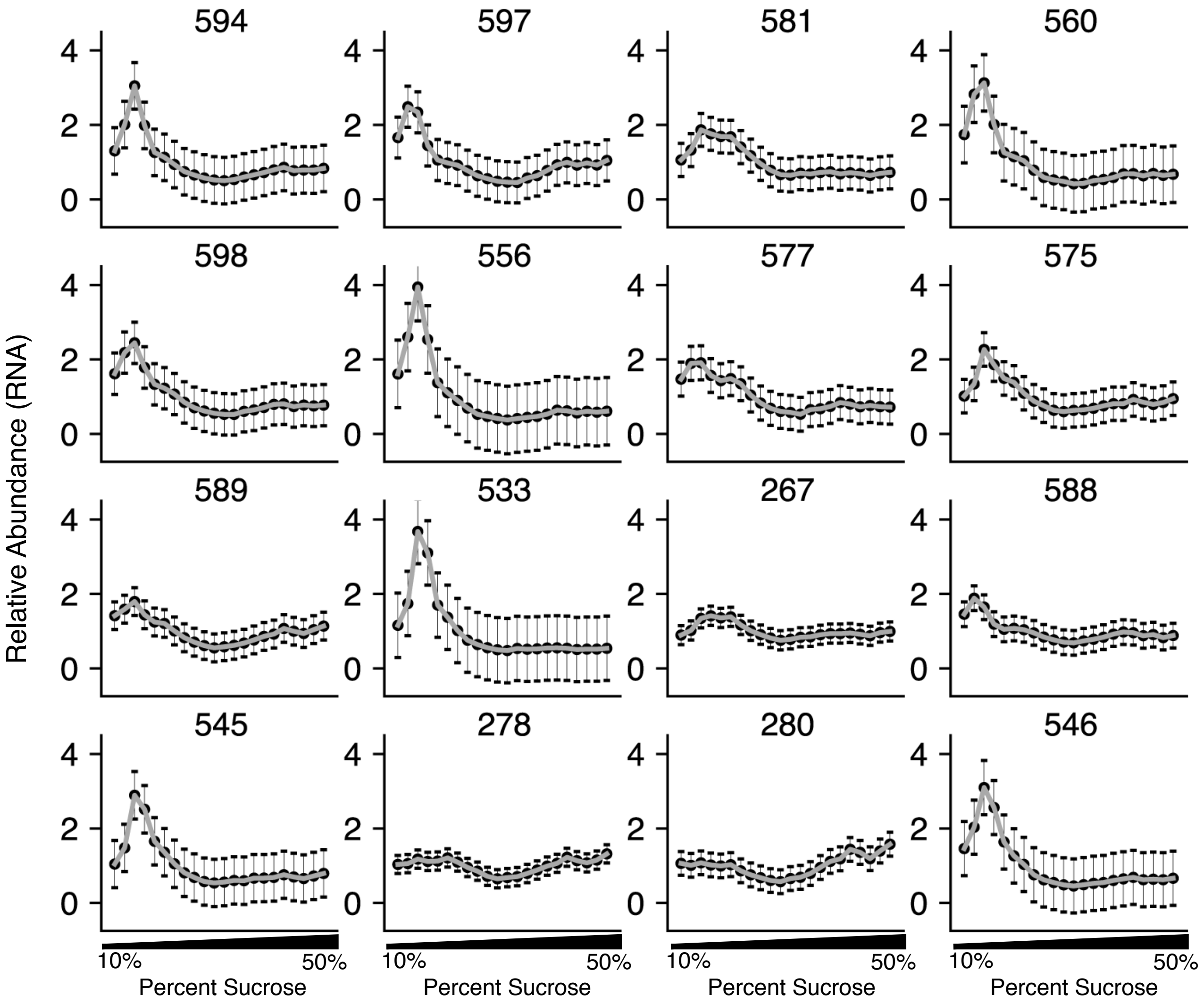

594 597 581 560
598 556 577 575
589 533 267 588
545 278 280 546
0 2 4
Relative Abundance (RNA)
10% 50%
Percent Sucrose

Figure S3

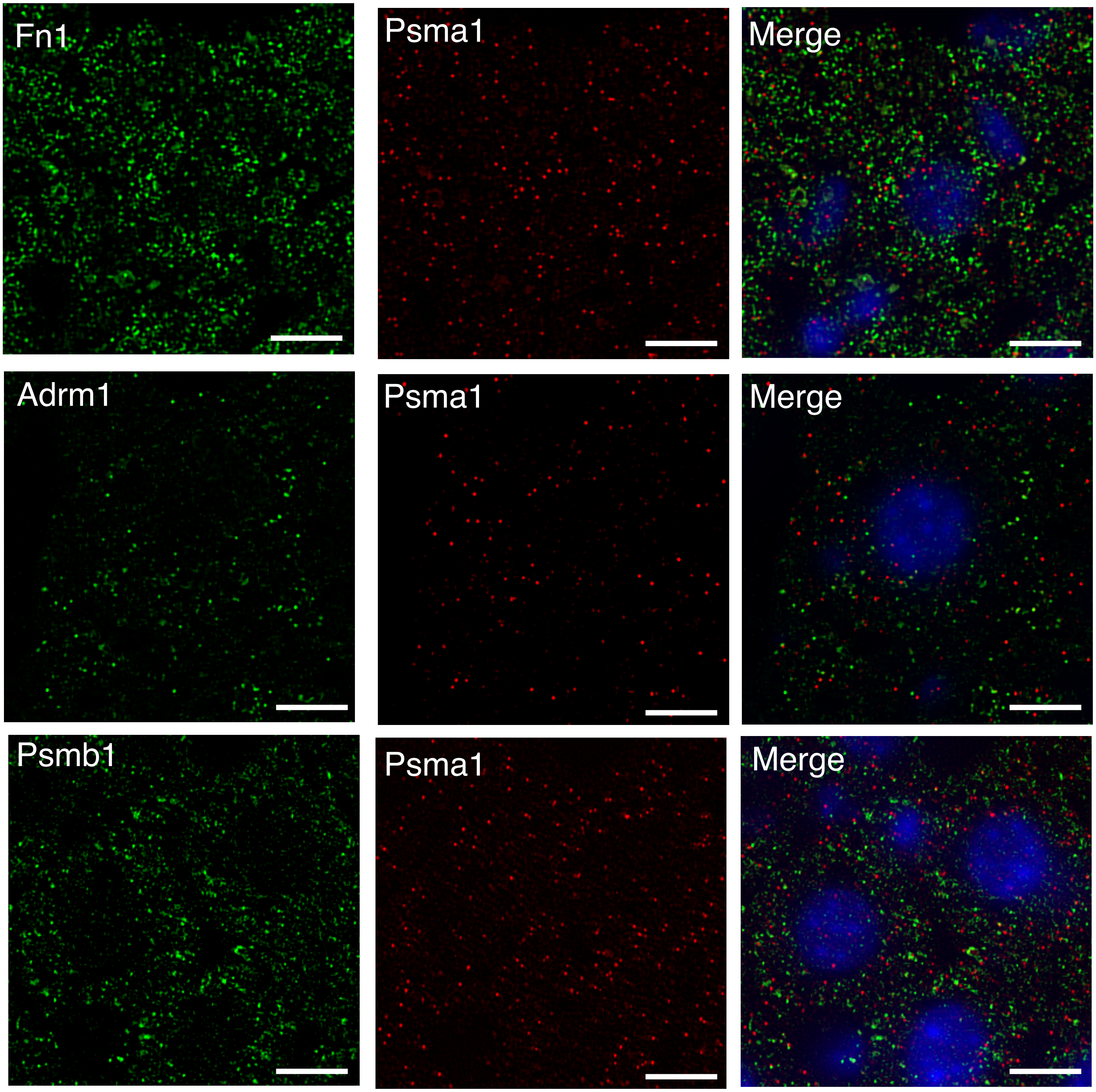

Figure S4

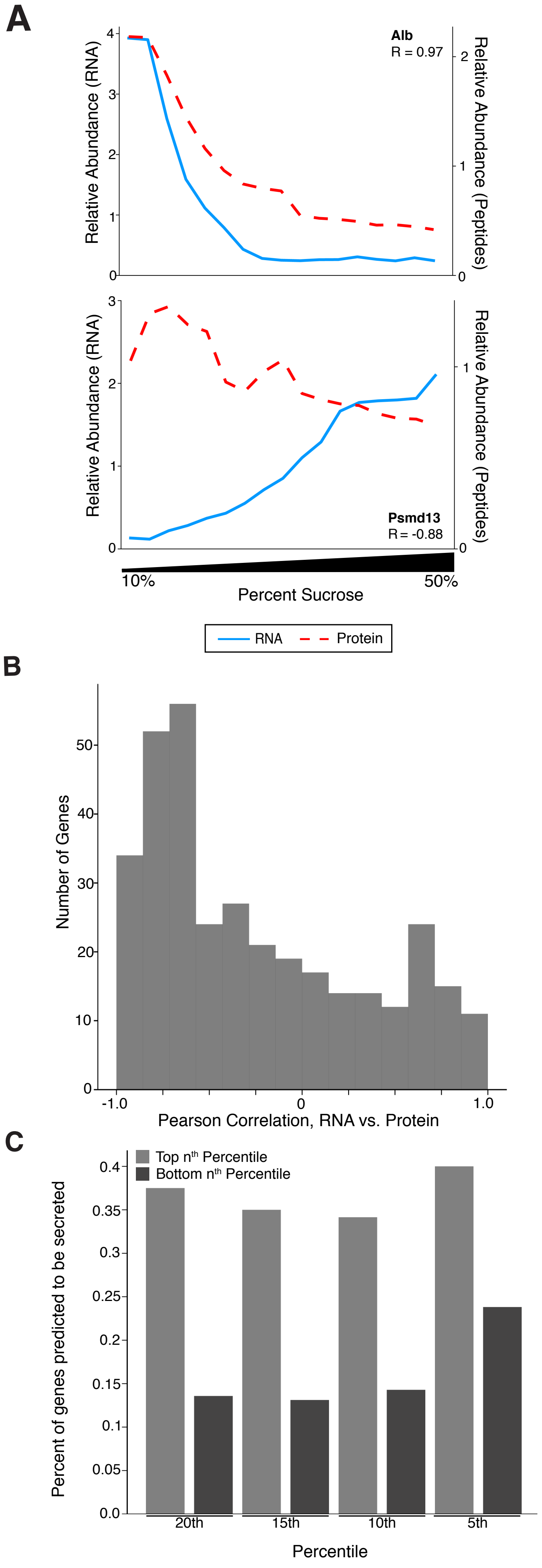
A
Alb
R = 0.97
Psmd13
R = -0.88
Relative Abundance (RNA)
Relative Abundance (Peptides)
10%
50%
Percent Sucrose
RNA
Protein
B
Number of Genes
Pearson Correlation, RNA vs. Protein
C
Top nth Percentile
Bottom nth Percentile
Percent of genes predicted to be secreted
20th
15th
10th
5th
Percentile

Figure S5

A

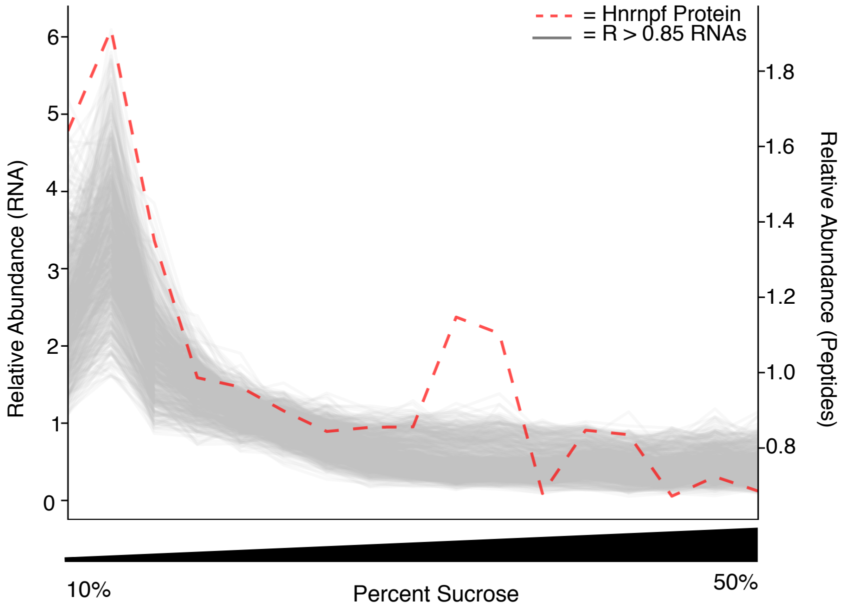

Correlated RNA GO Enrichment Categories

Cellular Compartment:
Derlin-1 retrotranslocation complex
cis-Golgi network
integral component of endoplasmic reticulum membrane
endoplasmic reticulum-Golgi intermediate compartment
endoplasmic reticulum lumen
rough endoplasmic reticulum

B

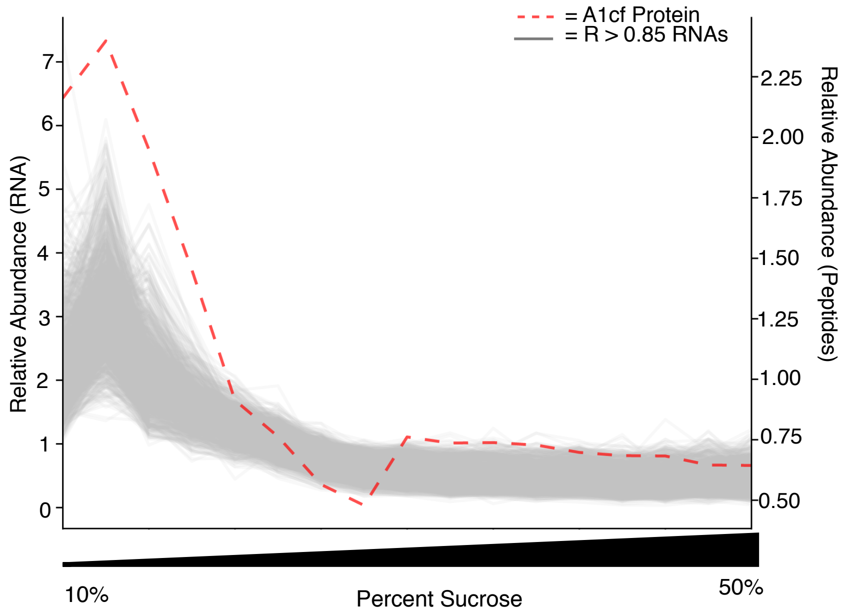

Correlated RNA GO Enrichment Categories

Cellular Compartment:
endoplasmic reticulum chaperone complex
endoplasmic reticulum-Golgi intermediate compartment
integral component of endoplasmic reticulum membrane
smooth endoplasmic reticulum
plasma lipoprotein particle
COPII-coated ER to Golgi transport vesicle

Figure S6

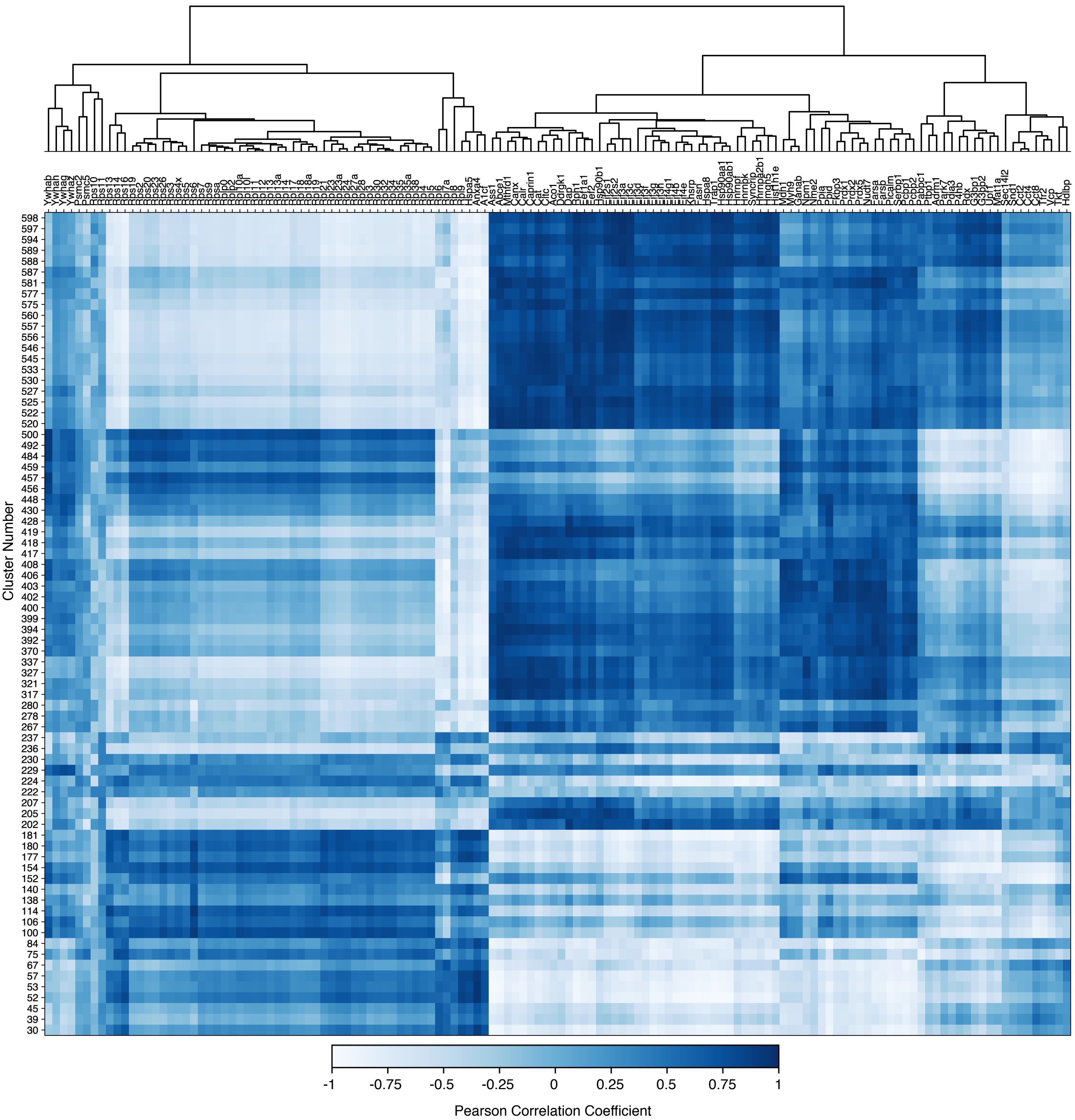
Cluster Number
-1
-0.75
-0.5
-0.25
0
0.25
0.5
0.75
1
Pearson Correlation Coefficient
